# Greater Intake of Pulses Is Associated with Lower Prevalence of Cardiometabolic Diseases among Adults in the United States: NHANES, 1999 to 2018

**DOI:** 10.1016/j.cdnut.2026.107716

**Published:** 2026-05-15

**Authors:** Tara Kamalakantha, Catherine Zavela, LuAnn K Johnson, Stephanie PB Caligiuri, Adam Drewnowski, Zach Conrad

**Affiliations:** 1 Department of Health Sciences, William & Mary, Williamsburg, VA, United States; 2 Global Research Institute, William & Mary, Williamsburg, VA, United States; 3 Independent Contractor, Warren, MN, United States; 4 Center for Public Health Nutrition, University of Washington, Seattle, WA, United States

**Keywords:** pulse, bean, lentil, cardiometabolic, cardiovascular, NHANES

## Abstract

**Background:**

Pulses, which include beans, peas, lentils, and chickpeas, are a cornerstone of dietary recommendations. However, studies evaluating the association between pulse intake and cardiometabolic diseases (CMD) have yielded mixed findings, and no study has evaluated this association in a nationally representative sample of United States adults. To address this gap, we evaluated the association between usual pulse intake and prevalent CMD among United States adults from 1999 to 2018, and analyses were stratified by CMD subtype and sociodemographic groups.

**Objectives:**

The objective was to evaluate the association between usual pulse intake and CMD prevalence in a nationally representative sample of United States adults.

**Methods:**

Data from 46,939 adults were acquired from the NHANES, 1999 to 2018. Usual pulse intake was estimated using the National Cancer Institute Method, and CMD outcomes were identified using standardized criteria. Multivariable logistic regression models were used to estimate the association between usual pulse intake and CMD outcomes, adjusting for demographic, behavioral, and dietary risk factors. All analyses were stratified by CMD subtype and sociodemographic group.

**Results:**

In fully adjusted models, each 1 oz/d increase in pulse intake was associated with a 19% lower prevalence of cardiometabolic disease overall [odds ratio (OR): 0.81, 95% confidence interval (CI): 0.68, 0.97; *P* = 0.026]. In sensitivity analyses that excluded BMI as a covariate, each 1 oz/d increase in pulse intake was associated with a 21% lower prevalence of CMD (OR: 0.79, 95% CI: 0.65, 0.95; *P* = 0.013) and a 20% lower prevalence of diabetes (OR: 0.80, 95% CI: 0.64, 0.99; *P* = 0.042). No differences were observed between sociodemographic groups for any outcome (*P* ≥ 0.05 for all comparisons).

**Conclusions:**

Greater pulse intake is associated with lower prevalence of CMD and subtypes. Exploratory analyses demonstrate no differences in the association of pulse intake with CMD or its subtypes across sociodemographic groups.

## Introduction

Cardiometabolic diseases (CMDs)—including heart disease, stroke, and diabetes—are leading causes of death in the United States, accounting for >4.8 million deaths from 1990 to 2017 [1]. By 2017 to 2018, only 6.8% of adults had optimal cardiometabolic health, as defined by meeting clinical standards for adiposity, blood glucose, blood lipids, blood pressure, and history of cardiovascular disease (CVD) [2]. This was down from 7.7% in 1999 to 2000 [2]. Major dietary interventions are urgently needed to dampen this trend. Suboptimal diet is the leading modifiable risk factor for CMD [3], which accounts for over 45% of all CMD deaths in the United States [4]. Key dietary contributors to CMD risk include a high intake of sodium, refined grains, and processed meats; and low consumption of pulses, whole grains, fruits, vegetables, and nuts and seeds [4].

Pulses, in particular, have been at the front of dietary recommendations. Pulses are a leguminous food group that includes beans, peas, lentils, and chickpeas. One additional serving of pulses was also associated with a 16% higher diet quality as measured by the Healthy Eating Index-2015 [5], which measures adherence to the Dietary Guidelines for Americans. In 2025, the United States Dietary Guidelines Advisory Committee recommended increasing daily pulse consumption from 1.5 to 2 cups per day (per 2000 kcal), and listed it above all other protein foods [6]. Pulses contain a number of bioactive compounds that have known associations with positive health outcomes, including fiber, resistant starch, protein, micronutrients, arginine, and polyphenols [[7], [8], [9]]. Data from the 2003 to 2014 NHANES showed that, among adults, pulse consumers had higher intakes of fiber, magnesium, and phosphorus compared with nonconsumers [10]. In a modeling study, others found that adding 1 serving of pulses to the average United States adult diet was associated with higher intakes of sodium, but also higher intakes of fiber, iron, magnesium, potassium, folate, and choline [5]. The low glycemic index and fermentable carbohydrate content of pulses can support glycemic control and satiety, whereas the fiber, antioxidants, and amino acids can help improve blood lipids, blood pressure, endothelial function, body weight regulation, and inflammation [[11], [12], [13]]. Polyphenols and antioxidant compounds in pulses may also help reduce oxidative stress, a contributor to chronic disease and premature mortality [14]. Consistent with these mechanisms, clinical studies have linked pulse consumption with improvements in glycemic control, lipid profiles, blood pressure, adiposity, and other cardiometabolic risk factors [[11], [12], [13]].

However, global meta-analyses of randomized controlled trials that evaluated the relationship of pulse intake with CMD outcomes and risk factors have yielded mixed findings [[15], [16], [17]]. Compared with the lowest level of pulse intake, Viguiliouk et al. [15] reported that the highest level of intake was associated with 8% lower incidence of hypertension, but no association was observed between pulse intake and CMD incidence and mortality. Hafiz et al. [13] reported that pulse intake was associated with lower postprandial glucose response when compared with other carbohydrate-rich foods, but long-term pulse intake was not associated with improved markers of glycemic control. More recently, Reyneke et al. [16] found no association between pulse consumption and systolic or diastolic blood pressure. Differences in study populations, exclusion criteria, and covariate adjustment may have contributed to these mixed findings. Country-level analyses with prespecified statistical controls for potential covariates are needed to better understand the association between pulse intake and CMD outcomes in specific contexts. Furthermore, these mixed findings from prior studies may also be attributed to a lack of subpopulation analyses, which are needed to identify group-level associations between pulse intake and CMD outcomes. This is particularly important because differences in pulse intake and related health outcomes may exist across sociodemographic groups. Educational attainment often shapes nutrition knowledge, income, and food purchasing behaviors [17]. Race and ethnicity may reflect differences in cultural food traditions, access to healthy foods, and chronic disease burden linked to structural inequities [[17], [18], [19]]. Sex differences may also be relevant because dietary patterns and cardiometabolic risk profiles often differ by sex [18,20]. Examining these groups can help determine whether the benefits of pulse intake are broadly shared or especially important for populations facing greater barriers to healthy eating.

To our knowledge, no studies have evaluated the association between pulse intake and multiple CMD outcomes in a nationally representative sample of United States adults. Addressing this gap will help clarify the association between pulse intake and cardiometabolic outcomes in the United States, which is an important component of nutrition surveillance and monitoring. To address this research need, we evaluated the association between usual intake of pulses and prevalent CMD, CVD, coronary artery disease (CAD), stroke, and diabetes among United States adults from 1999 to 2018. Outcomes were evaluated overall and stratified by sociodemographic characteristics to identify populations most likely to benefit from targeted interventions.

## Methods

### Data acquisition

Data on individual-level intake of food and nutrients, sociodemographic characteristics, and prevalent CAD, stroke, and diabetes were acquired from the NHANES, 1999 to 2018. NHANES uses a stratified, clustered, multistage sampling design to collect data from ∼5000 noninstitutionalized participants per year. Data are collected from participants by trained staff using in-person surveys, physical examinations, and laboratory tests. Some population groups are oversampled to increase reliability and precision for subgroup analysis [21]. Data are collected continuously but released in 2-y cycles [22]. Dietary data are collected from participants using an in-person 24-h dietary recall, and a subsequent recall is administered by telephone 3 to 10 d later on ∼80% of the sample. The computer-assisted Automated Multiple Pass Method is used to minimize respondent burden and increase reliability and validity [23]. The NHANES data collection protocol was approved by the National Center for Health Statistics (NCHS) Ethics Review Board, and all participants provided written informed consent. The present study is a secondary analysis of publicly available and deidentified data and was deemed exempt from human studies ethical review by the Institutional Review Board at William & Mary.

### Dietary data

As part of the 24-h dietary recall procedure, participants provide information on the amount of every food and beverage (hereafter, *food*) consumed over the preceding 24-h period, which is converted from their reported units to gram weight. In collaboration with NCHS, USDA assigns a unique text description and 8-digit food code to each food, which are based on the predominant ingredient in each food, using the coding scheme established by the USDA Food and Nutrient Database for Dietary Studies [24]. USDA staff also use automated procedures to match each of these foods to supplementary databases that provide information on the amount of nutrients (USDA FoodData Central [25]) and food groups [USDA Food Patterns Equivalents Database (FPED) [26]] contained within each food. Data on food groups are converted to food pattern equivalents such as ounce-equivalents, cup-equivalents, and teaspoon-equivalents. For example, the FPED was used to estimate the amount of pulses present in different mixed dishes, and to convert this amount from grams to ounce-equivalents. These data are also aggregated to the person level and are available as downloadable files from the NHANES website (nutrient intake) [27] and a website maintained by the USDA Agricultural Research Service (intake of food groups) [26].

Using the data files described above, a 2-step procedure was used to estimate the quantity of pulses consumed by each participant. First, all foods that contained pulses were identified based on their text descriptions, which resulted in 339 pulse-containing foods (beans = 279 foods, chickpeas = 20 foods, lentils = 17 foods, and peas = 23 foods). Second, for each food identified in the previous step, the USDA FPED [26] was used to convert the amount consumed in grams to ounce-equivalents of legumes.

### Outcome ascertainment

The American Heart Association diagnostic criteria for CMD were used to identify prevalent cases [28]. Prevalent diabetes was identified by self-report of physician diagnosis (NHANES variable name: DIQ010), fasting plasma glucose ≥126 mg/dL (LBXGLU), or self-report of current prescription drug treatment (DIQ050 or DIQ070). Participants with a history of stroke (MCQ160F) were identified by self-report of physician diagnosis. Prevalent CAD was identified by self-report of physician diagnosis of myocardial infarction (MCQ160C or MCQ160E) or angina (MCQ160D) or self-report of taking angina medications nitroglycerin, isosorbide dinitrate, or isosorbide mononitrate (RDXDRUG), and undiagnosed angina was based on the Rose questionnaire (CDQ001-CDQ009G). Participants with CAD or a history of stroke were identified as having CVD, and participants with CMD included those with CVD or diabetes.

### Statistical analyses

The National Cancer Institute’s (NCI) usual intake methodology was used to assess the usual intake of foods, food groups, and nutrients [29]. The NCI method uses the SAS macros (National Cancer Institute (NCI)) MIXTRAN, DISTRIB, and INDIVINT to estimate within-person variation of the entire sample using data from two 24-h recalls collected from most participants [[30], [31], [32]]. MIXTRAN fits a nonlinear mixed effects model to repeated 24-h recalls, and parameter estimates are passed to DISTRIB to estimate the distribution of usual intake in the sample. Parameter estimates from MIXTRAN are also passed to INDIVINT, which estimates intakes at the individual level, which are used for regression modeling.

Multivariable logistic regression models assessed the linear association between pulse intake and each binary outcome: prevalent CMD, CVD, CAD, stroke, and diabetes. Model 1 was adjusted for age (20–30, 31–50, 51–60, 61–70, or ≥71 y) and gender (male or female). Model 2 was adjusted for model 1 plus sociodemographic and behavioral risk variables, including education (< high school, high school or equivalent, some college, or college graduate), race-ethnicity (non-Hispanic White, non-Hispanic Black, Mexican-American, or other), income-to-poverty ratio (<0.75, 0.75–1.30, 1.31–1.99, 2.00–3.99, or ≥4.00), physical activity (sedentary, moderate, or vigorous), smoking status (<100 cigarettes in lifetime, ≥100 cigarettes in lifetime but not current smoker, ≥100 cigarettes in lifetime and currently smoke some days, or ≥100 cigarettes in lifetime and currently smoke everyday), BMI (kg/m^2^; <18.5, 18.5–<25, 25–<30, ≥30), and survey cycle (continuous). Model 3 was adjusted for model 2 plus diet risk variables, including energy intake (continuous), refined grains (continuous), added sugars (continuous), saturated fat (continuous), alcohol (continuous), sodium (continuous), fruit (continuous), vegetables (continuous), and whole grains (continuous). Missing values for each nondietary covariate were included as a dummy indicator to preserve sample size. To explore potential effect modification of the sociodemographic variables, an interaction term for age, gender, education, and income-to-poverty ratio was iteratively included in stratified models. We included BMI in models 2 and 3 as a potential confounder because, conceptually, it is independently associated with the exposure (pulse intake) and outcomes (cardiometabolic conditions). At the same time, it is also plausible that BMI lies on the causal pathway between the exposure and outcome. Therefore, our main analyses modeled BMI as a confounder, and sensitivity analyses were used to examine whether removing BMI from models 2 and 3 meaningfully changed the results.

Statistical significance was set at *P* < 0.05. SEs were estimated using the balanced repeated replication method while accounting for the multistage probability sampling design of the NHANES. Confidence intervals (CIs) and *P* values for odds ratios (ORs) were calculated using the NCI macro BRR_PVALUE_CI (v1.1) [30]. Stata 16.1 (StataCorp) was used for data management, and SAS 9.4 (SAS Institute) was used for all analyses.

## Results

A total of 96,766 participants provided dietary data from 1999 to 2018. Participants were excluded if they were aged <20 y (*n =* 44,368); pregnant or breastfeeding (*n =* 1827); had incomplete or unreliable dietary recalls as deemed by trained NHANES staff (*n =* 3570); or had incomplete data on CMD status (*n =* 62). A total of 46,939 participants were included in the analytic sample. The majority of participants were aged 31 to 70 y (68%), had income-to-poverty ratios ≥2.00 (60%), had at least some college education (59%), and were non-Hispanic White (69%; Supplemental Table 1).

**FIGURE 1:**
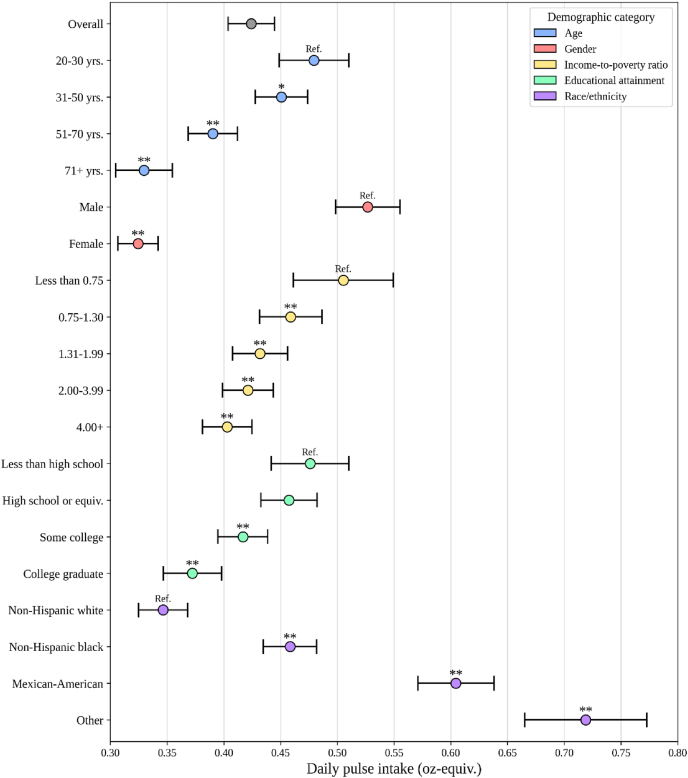
Mean daily pulse intake by sociodemographic characteristics (*n* = 46,939). Horizontal bars indicate 95% CI. Within each sociodemographic category, differences between the first group and the other groups were tested using Wald tests (∗<0.05, ∗∗<0.01), with Bonferroni correction for multiple comparisons. All results were adjusted for energy intake and survey cycle. CI, confidence interval.

Mean daily pulse intake for the entire sample was 0.42 oz-equivalents (Figure 1). Intake was higher in younger age groups (*P <* 0.05), males (*P <* 0.01), lower income groups (*P <* 0.01), and non-White groups (*P <* 0.01). Those with less than high school educational attainment had greater pulse intake than those with at least some college (*P <* 0.01). Median pulse intake for the entire sample was 0.25 oz-equivalents, with an IQR of 0.11 to 0.53 oz-equivalents (Supplemental Figure 1). Median intakes ranged from 0.21 oz-equivalents for non-Hispanic Whites (IQR: 0.10–0.43 oz-equivalents) and females (IQR: 0.10–0.42 oz-equivalents) to 0.50 for other race/ethnicities (IQR: 0.24–0.94 oz-equivalents).

In models adjusted for age and sex (model 1), each additional 1 oz/d of pulse intake was associated with 29% lower odds of CVD (OR: 0.71; 95% CI: 0.57, 0.88; *P =* 0.002), 27% lower odds of CAD (OR: 0.73, 95% CI: 0.59, 0.92; *P =* 0.007), and 40% lower odds of stroke (OR: 0.60, 95% CI: 0.39, 0.92; *P =* 0.019; Table 1). In model 2, which was further adjusted for sociodemographic and behavioral risk variables, the findings for CVD and stroke were attenuated but remained statistically significant. In model 3, which was further adjusted for dietary variables, greater pulse intake was associated with 19% lower odds of CMD (OR: 0.81; 95% CI: 0.67, 0.97; *P =* 0.026). In sensitivity analyses (Supplemental Table 2), removing BMI from model 2 was associated with statistically significant findings for CMD (*P =* 0.021), CVD (*P =* 0.015), CAD (*P =* 0.041), and stroke (*P =* 0.040); and in model 3, statistical significance was detected for CMD (*P =* 0.013) and diabetes (*P =* 0.042).

**TABLE 1:** Association between usual pulse intake and cardiometabolic disease prevalence (*n* = 46,939).

Prevalence outcome	OR (95% CI)^1^		*P* value
Model 1^2^			
CMD	0.89	(0.74, 1.08)	0.239
CVD	0.71	(0.57, 0.88)	0.002
CAD	0.73	(0.59, 0.92)	0.007
Stroke	0.60	(0.39, 0.92)	0.019
Diabetes	1.07	(0.87, 1.32)	0.506
Model 2^3^			
CMD	0.82	(0.68, 1.00)	0.050
CVD	0.77	(0.62, 0.96)	0.020
CAD	0.80	(0.63, 1.00)	0.054
Stroke	0.64	(0.41, 0.98)	0.042
Diabetes	0.90	(0.73, 1.11)	0.331
Model 3^4^			
CMD	0.81	(0.67, 0.97)	0.026
CVD	0.83	(0.66, 1.03)	0.094
CAD	0.82	(0.65, 1.05)	0.112
Stroke	0.75	(0.48, 1.19)	0.225
Diabetes	0.82	(0.65, 1.02)	0.072

Abbreviations: CAD, coronary artery disease; CI, confidence interval; CMD, cardiometabolic disease; CVD, cardiovascular disease; OR, odds ratio.

1 OR associated with every once ounce-equivalent increase in usual pulse intake.

2 Adjusted for age and sex.

3 Adjusted for model 1 + sociodemographic and behavioral risk factors.

4 Adjusted for model 2 + diet risk variables.

Model 3 was further stratified by sociodemographic characteristics, and the results are presented in TABLE 2, TABLE 3, TABLE 4, TABLE 5, TABLE 6. Every 1 oz/d increase in pulse intake was associated with lower odds of prevalent cardiometabolic outcomes for some groups (CMD among males and non-Hispanic Whites; CVD among college graduates; CAD among non-Hispanic Blacks; and stroke among participants with an income-to-poverty ratio of 0.75–1.30). However, no differences were observed between sociodemographic groups for any outcome (*P* ≥ 0.05 for all comparisons).

**TABLE 2:** Association between usual pulse intake and cardiometabolic disease prevalence by socioeconomic characteristics (*n* = 46,939).

Characteristic	OR (95% CI)^1^		*P* value^2^	*P*-difference^3^
Gender				
Female	0.91	(0.66, 1.26)	0.575	Reference
Male	0.75	(0.58, 0.97)	0.030	0.030
Age (y)				
20–30	0.38	(0.11, 1.27)	0.114	Reference
31–50	0.80	(0.54, 1.18)	0.250	0.300
51–70	0.83	(0.63, 1.08)	0.155	0.200
71+	0.87	(0.60, 1.28)	0.484	0.218
Education				
Less than high school	0.80	(0.58, 1.09)	0.155	Reference
High school or equivalent	0.89	(0.59, 1.35)	0.584	0.690
Some college	0.94	(0.61, 1.44)	0.771	0.558
College graduate	0.62	(0.40, 0.96)	0.031	0.374
Race/ethnicity				
Non-Hispanic White	0.73	(0.53, 1.00)	0.047	Reference
Non-Hispanic Black	1.05	(0.63, 1.76)	0.845	0.218
Mexican-American	0.80	(0.58, 1.11)	0.183	0.660
Other	0.93	(0.63, 1.36)	0.700	0.355
Income-to-poverty ratio				
<0.75	0.80	(0.60, 1.08)	0.138	Reference
0.75–1.30	0.77	(0.55, 1.07)	0.119	0.866
1.31–1.99	0.91	(0.63, 1.33)	0.633	0.495
2.00–3.99	0.80	(0.60, 1.06)	0.120	0.992
4.00+	0.81	(0.56, 1.15)	0.235	0.974

Abbreviations: CI, confidence interval; OR, odds ratio.

1 OR associated with a 1-oz-equivalent increase in usual pulse intake. The model for each sociodemographic characteristic was adjusted for the other characteristics (age, gender, education, race-ethnicity, income-to-poverty ratio, and physical activity), smoking status, BMI, survey cycle, and intake of energy, refined grains, added sugars, saturated fat, alcohol, sodium, fruit, vegetables, and whole grains.

2 Test if the OR is significantly different from 1.

3 Within each characteristic, a test of difference between the reference group and each of the other groups.

**TABLE 3:** Association between usual pulse intake and cardiovascular disease prevalence by socioeconomic characteristics (*n* = 46,939).

Characteristic	OR (95% CI)^1^		*P* value^2^	*P*-difference^3^
Gender				
Female	1.00	(0.62, 1.62)	0.987	Reference
Male	0.74	(0.54, 1.01)	0.061	0.061
Age (y)				
20–30	0.73	(0.13, 4.14)	0.716	Reference
31–50	0.65	(0.38, 1.09)	0.103	0.900
51–70	0.81	(0.59, 1.10)	0.176	0.902
71+	1.07	(0.72, 1.60)	0.728	0.671
Education				
Less than high school	0.81	(0.55, 1.21)	0.300	Reference
High school or equivalent	1.21	(0.79, 1.86)	0.375	0.170
Some college	0.89	(0.59, 1.33)	0.557	0.761
College graduate	0.46	(0.23, 0.91)	0.026	0.193
Race/ethnicity				
Non-Hispanic White	0.86	(0.63, 1.19)	0.364	Reference
Non-Hispanic Black	0.79	(0.48, 1.29)	0.341	0.761
Mexican-American	0.74	(0.48, 1.15)	0.180	0.585
Other	0.80	(0.50, 1.31)	0.375	0.804
Income-to-poverty ratio				
<0.75	0.98	(0.64, 1.49)	0.905	Reference
0.75–1.30	0.83	(0.52, 1.34)	0.439	0.660
1.31–1.99	1.03	(0.62, 1.70)	0.910	0.853
2.00–3.99	0.75	(0.54, 1.05)	0.090	0.348
4.00+	0.78	(0.52, 1.19)	0.251	0.447

Abbreviations: CI, confidence interval; OR, odds ratio.

1 OR associated with a 1-oz-equivalent increase in usual pulse intake. The model for each sociodemographic characteristic was adjusted for the other characteristics (age, gender, education, race-ethnicity, income-to-poverty ratio, and physical activity), smoking status, BMI, survey cycle, and intake of energy, refined grains, added sugars, saturated fat, alcohol, sodium, fruit, vegetables, and whole grains.

2 Test if the OR is significantly different from 1.

3 Within each characteristic, a test of difference between the reference group and each of the other groups.

**TABLE 4:** Association between usual pulse intake and coronary artery disease prevalence by socioeconomic characteristics (*n* = 46,939).

Characteristic	OR (95% CI)^1^		*P* value^2^	*P*-difference^3^
Gender				
Female	1.06	(0.69, 1.62)	0.800	Reference
Male	0.72	(0.52, 1.00)	0.050	0.050
Age, y				
20–30	0.21	(0.01, 6.56)	0.375	Reference
31–50	0.56	(0.32, 1.03)	0.064	0.575
51–70	0.81	(0.58, 1.12)	0.201	0.445
71+	1.17	(0.74, 1.84)	0.502	0.336
Education				
Less than high school	0.76	(0.52, –1.12)	0.170	Reference
High school or equivalent	1.25	(0.77, 2.01)	0.363	0.121
Some college	0.81	(0.51, 1.27)	0.351	0.856
College graduate	0.54	(0.22, 1.31)	0.172	0.515
Race/ethnicity				
Non-Hispanic White	0.86	(0.61, 1.21)	0.387	Reference
Non-Hispanic Black	0.52	(0.28, 0.96)	0.037	0.147
Mexican-American	0.77	(0.49, 1.23)	0.279	0.705
Other	0.90	(0.57, 1.43)	0.660	0.869
Income-to-poverty ratio				
<0.75	0.82	(0.52, 1.31)	0.408	Reference
0.75–1.30	0.75	(0.51, 1.10)	0.144	0.781
1.31–1.99	0.98	(0.58, 1.63)	0.922	0.621
2.00–3.99	0.85	(0.57, 1.26)	0.408	0.926
4.00+	0.83	(0.54, 1.27)	0.394	0.977

Abbreviations: CI, confidence interval; OR, odds ratio.

1 OR associated with a 1-oz-equivalent increase in usual pulse intake. The model for each sociodemographic characteristic was adjusted for the other characteristics (age, gender, education, race-ethnicity, income-to-poverty ratio, and physical activity), smoking status, BMI, survey cycle, and intake of energy, refined grains, added sugars, saturated fat, alcohol, sodium, fruit, vegetables, and whole grains.

2 Test if the OR is significantly different from 1.

3 Within each characteristic, a test of difference between the reference group and each of the other groups.

**TABLE 5:** Association between usual pulse intake and stroke prevalence by socioeconomic characteristics (*n* = 46,939).

Characteristic	OR (95% CI)^1^		*P* value^2^	*P*-difference^3^
Gender				
Female	0.75	(0.33, 1.69)	0.482	Reference
Male	0.76	(0.42, 1.38)	0.366	0.366
Age (y)				
20–30	1.40	(0.16, 12.66)	0.761	Reference
31–50	0.59	(0.19, 1.81)	0.354	0.502
51–70	0.66	(0.34, 1.27)	0.213	0.502
71+	0.95	(0.52, 1.74)	0.866	0.721
Education				
Less than high school	1.01	(0.55, 1.85)	0.987	Reference
High school or equivalent	1.08	(0.48, 2.42)	0.848	0.885
Some college	0.68	(0.34, 1.36)	0.275	0.399
College graduate	0.22	(0.05, 1.07)	0.060	0.052
Race/ethnicity				
Non-Hispanic White	0.83	(0.43, 1.62)	0.589	Reference
Non-Hispanic Black	0.85	(0.19, 3.82)	0.832	0.981
Mexican-American	0.67	(0.29, 1.57)	0.353	0.692
Other	0.54	(0.20, 1.44)	0.215	0.415
Income-to-poverty ratio				
<0.75	1.37	(0.83, 2.27)	0.221	Reference
0.75–1.30	0.77	(0.36, 1.65)	0.498	0.245
1.31–1.99	1.13	(0.58, 2.21)	0.723	0.630
2.00–3.99	0.50	(0.24, 1.03)	0.058	0.015
4.00+	0.51	(0.22, 1.20)	0.124	0.037

Abbreviations: CI, confidence interval; OR, odds ratio.

1 OR associated with a 1-oz-equivalent increase in usual pulse intake. The model for each sociodemographic characteristic was adjusted for the other characteristics (age, gender, education, race-ethnicity, income-to-poverty ratio, and physical activity), smoking status, BMI, survey cycle, and intake of energy, refined grains, added sugars, saturated fat, alcohol, sodium, fruit, vegetables, and whole grains.

2 Test if the OR is significantly different from 1.

3 Within each characteristic, a test of difference between the reference group and each of the other groups.

**TABLE 6:** Association between usual pulse intake and diabetes prevalence by socioeconomic characteristics (*n* = 46,939).

Characteristic	OR (95% CI)^1^		*P* value^2^	*P*-difference^3^
Gender				
Female	0.80	(0.58, 1.09)	0.158	Reference
Male	0.83	(0.63, 1.08)	0.160	0.1601
Age (y)				
20–30	0.28	(0.06, 1.23)	0.091	Reference
31–50	0.83	(0.45, 1.54)	0.551	0.236
51–70	0.82	(0.59, 1.14)	0.229	0.147
71+	0.93	(0.59, 1.46)	0.736	0.150
Education				
Less than high school	0.82	(0.60, 1.12)	0.209	Reference
High school or equivalent	0.76	(0.47, 1.23)	0.259	0.790
Some college	0.89	(0.54, 1.43)	0.605	0.800
College graduate	0.78	(0.46, 1.33)	0.361	0.888
Race/ethnicity				
Non-Hispanic White	0.70	(0.47, 1.05)	0.085	Reference
Non-Hispanic Black	1.06	(0.62, 1.81)	0.829	0.235
Mexican-American	0.84	(0.59, 1.20)	0.344	0.467
Other	0.96	(0.65, 1.42)	0.845	0.273
Income-to-poverty ratio				
<0.75	0.76	(0.55, 1.05)	0.089	Reference
0.75–1.30	0.68	(0.52, 0.89)	0.006	0.636
1.31–1.99	0.79	(0.52, 1.20)	0.260	0.861
2.00–3.99	0.99	(0.72, 1.34)	0.922	0.254
4.00+	0.86	(0.58, 1.27)	0.435	0.639

Abbreviations: CI, confidence interval; OR, odds ratio.

1 OR associated with a 1-oz-equivalent increase in usual pulse intake. The model for each sociodemographic characteristic was adjusted for the other characteristics (age, gender, education, race-ethnicity, income-to-poverty ratio, and physical activity), smoking status, BMI, survey cycle, and intake of energy, refined grains, added sugars, saturated fat, alcohol, sodium, fruit, vegetables, and whole grains.

2 Test if the OR is significantly different from 1.

3 Within each characteristic, a test of difference between the reference group and each of the other groups.

## Discussion

In this nationally representative study of nearly 47,000 adults over 20 y, each additional 1 oz-equivalent of daily pulse intake was associated with 19% lower prevalence of CMD after adjustment for sociodemographic, behavioral, and dietary risk factors. No differences in the association of pulse intake with CMD or its subtypes were observed between sociodemographic groups. To our knowledge, this is the first study to investigate the association between pulse intake and multiple CMD outcomes in a nationally representative sample of United States adults.

Although we found that greater pulse intake was associated with lower prevalence of CMD overall, this association was not observed for CMD subtypes (CVD, CAD, stroke, and diabetes). The number of CMD cases represents the sum of its subtypes, which provides greater statistical power to detect statistical significance than for each subtype separately. These findings contribute to a body of literature that shows beneficial or null associations of pulse intake with CMD and associated risk factors. In global meta-analyses, greater pulse intake was associated with improved markers of postprandial glycemic control, but long-term pulse intake was not [13]. Other global meta-analyses indicate conflicting associations between pulse intake and blood pressure [15,16], and no association with CMD incidence or mortality [15]. Some of these discrepancies may be attributed to unmeasured differences across geographic regions or nonspecific exclusion criteria, which makes it challenging to discern any differential impact of confounding variables across individual studies. Greater research efforts are needed to understand additional sources of these discrepancies.

Scientists report variable findings from controlled clinical trials of pulse intake on cardiometabolic risk factors. For example, in a 12-wk clinical trial, 38 participants with elevated waist circumference and serum triglyceride levels consumed a lentil-rich diet (140 g of green lentils/d) or a meat-based diet [33]. Consumption of lentils resulted in reduced fasting LDL cholesterol and total cholesterol, but also reduced HDL cholesterol, and no changes in serum triglycerides or markers of glycemic control were observed [33]. Among females with diagnosed polycystic ovary syndrome who completed a 16-wk intervention consisting of a high-pulse healthy diet (90–225 g or 2–5 oz), HDL cholesterol increased after the trial period compared with baseline and remained high after 12-mo follow-up [34]. Markers of glycemic control and systolic blood pressure were lower after the 16-wk intervention compared with baseline, but were not maintained after 1 y [34]. Scientists have also investigated the effects of pulse intake on physiological parameters of healthy individuals. In 1 acute feeding trial, 0.25 to 0.5 cups (1–2 oz) of beans was associated with lower postprandial glucose and insulin response compared with equal portions of corn (62%–70% lower), rice (32%–37% lower), macaroni (36%–42% lower), or potato (45% lower) [35]. This is consistent with others, who found that substituting lentils for half of the carbohydrates from white rice or potato was associated with lower postprandial glucose and insulin response than consuming only white rice or potato [36]. Further research is needed to understand whether different pulse types have different associations with chronic disease and to understand whether the health benefits of greater pulse intake may differ between healthy and at-risk populations.

Sustained dietary change is most effective when it extends beyond education to also address the social and environmental contexts that shape food choice and adoption of healthy habits [37,38]. Interventions that incorporate pulse-forward meal framing, accessible recipes, specific substitution guidance, expanded availability, and integration into food assistance programs may improve adherence and diet quality in diverse populations. These approaches acknowledge that food selection is influenced not only by knowledge but also by availability, social norms, and perceived convenience. Evidence from dietary interventions that focus on nonpulse foods supports the cardiometabolic relevance of strategies based on these principles. In a long-term clinical trial conducted in a primarily male cohort, 30 g of ground flaxseed was incorporated into foods that had been tested in focus groups to ensure acceptability and desirability for the target population, with additional products provided to spouses to support adherence. This approach achieved high adherence over 1 y and produced significant reductions in systolic blood pressure, diastolic blood pressure, and blood cholesterol [[39], [40], [41], [42]]. In a longitudinal cohort of older men, participants were able to incorporate greater intake within the meat and alternatives group, including pulses, and this achievable dietary shift was associated with improved self-rated health [43]. Collectively, these findings indicate that when dietary strategies prioritize food acceptability and supportive environments, sustained adherence is achievable and can lead to meaningful cardiometabolic benefits.

Extending these principles beyond controlled and cohort settings to the broader population requires alignment with contemporary food environments that influence daily food choices. Online platforms are now a major source of nutrition information [44,45], and scientists illustrate that dietary behaviors are shaped by peer norms, digital food environments, and repeated exposure to food messaging [46]. Positioning pulses within familiar and socially endorsed eating patterns through peer modeling, evidence-based influencer content, and campus or workplace initiatives may improve acceptability. For men, frameworks such as the Health Belief Model indicate that emphasizing tangible benefits and perceived behavioral control is central to effective messaging [47], suggesting that framing pulses in terms of affordability, convenience, and advantages for cardiometabolic health and physical performance may be more effective than risk-focused communication. Collectively, these observations support a strategy that integrates targeted behavioral messaging with supportive environmental change. Communicating the benefits of pulses in ways that align with outcomes that are personally relevant to the populations of interest, together with increasing the access and visibility of pulse-rich foods, represents a plausible pathway for improving intake and may contribute to downstream reductions in mortality risk.

This study has several strengths. Analyses were stratified by CMD subtypes (CVD, CAD, stroke, and diabetes) and sociodemographic characteristics (age, sex, income level, educational attainment, and race-ethnicity), which supports population-level monitoring and surveillance efforts. Usual dietary intakes and their association with CMD prevalence were estimated using the NCI method, which reduces bias by accounting for interindividual and intraindividual variation and correlating the intake amount to the probability of intake [29]. All statistical models were adjusted for known risk factors related to sociodemographic, behavioral, and dietary variables. Finally, the sampling design and large sample make these findings generalizable to the United States adult population.

This study also has several limitations. The cross-sectional design prevents causal inference, so it is possible that some participants modified their diets after CMD diagnosis. Other study designs are needed to confirm directionality and estimate dose–response relationships. Although prior research has demonstrated the health benefits of individual pulse types [33,35,36,48], comparative studies are needed to understand whether these have different associations with cardiometabolic health outcomes. Self-reported dietary data are subject to social desirability bias, which may have introduced measurement error, but these data can still provide a useful estimation of population-level dietary intake [49]. Finally, all statistical models included a robust set of potential confounding variables, but residual confounding cannot be ruled out.

In conclusion, this large, nationally representative study found that adults who consumed just 1 additional oz-equivalent of pulses per day had a 19% lower prevalence of CMD. Exploratory analyses demonstrated no differences in the association of pulse intake with CMD or its subtypes across sociodemographic groups. Pulse intake across all groups remained far below recommended amounts, suggesting an opportunity for improvement through culturally tailored interventions in clinical and public health settings. Further research is needed to understand the association of individual pulse types with chronic disease outcomes in United States populations.

## Author contributions

The authors’ responsibilities were as follows – ZC, AD: designed the research; ZC, LKJ: conducted the research, provided essential materials, and analyzed data; TK, CZ, SPBC, ZC: wrote the paper; ZC: responsible for its final content; and all authors: read and approved the final manuscript.

## Data availability

The data described in the manuscript are publicly available at https://www.cdc.gov/nchs/nhanes/index.html. Data described in the manuscript, code book, and analytic code will be made available upon request, pending review.

## Declaration of Generative AI and AI-Assisted Technologies in the Writing Process

The authors declare that no generative AI or AI-assisted technologies were used in the writing of this manuscript.

## Funding

Financial support was received from the USDA ARS FY 2023 Pulse Crop Health Initiative (58-3060-3-050). The funder had no role in the design, implementation, analysis, interpretation of the data, manuscript preparation, or the decision to submit the manuscript for publication.

## Conflict of interest

ZC has research funding from the Jeffress Trust Awards Program for Research Advancing Health Equity, American Pistachio Growers, National Dairy Council, and National Pork Board. AD is the original developer of the Naturally Nutrient Rich and the Nutrient-Rich Food nutrient profiling models and is a member of scientific advisory panels for Nestlé, Lesaffre, and BEL. AD has worked with Ajinomoto, Carbohydrate Quality Panel supported by Potatoes USA, dsm-firmenich, FoodMinds, FrieslandCampina Institute, Kraft Heinz, Meiji, MS-Nutrition, National Pork Board, Nutrition Impact LLC, Nutrition Institute, PepsiCo, Samsung, and Soremartec on quantitative ways to assess nutrient density of foods. The University of Washington receives grants and contracts from public and private sources.
