# Supplementary material for: Greater Intake of Pulses Is Associated with Lower Prevalence of Cardiometabolic Diseases among Adults in the United States: NHANES, 1999 to 2018
Source: Curr Dev Nutr. 2026 May 15;10(6):107716. doi: 10.1016/j.cdnut.2026.107716 (PMC13262142; doi:10.1016/j.cdnut.2026.107716)
Supplement: Multimedia component 1 [file mmc1.pdf]

Supplementary Materials

Greater intake of pulses is associated with lower prevalence of cardiometabolic diseases among adults in the United States: National Health and Nutrition Examination Survey, 1999-2018

Tara Kamalakantha et al.

Supplemental Table 1: Demographic characteristics of study participants, 1999-2018 (n=46,939)

Supplemental Table 2: Association between usual pulse intake and cardiometabolic disease prevalence, without adjustment for Body Mass Index (n=46,939)

Supplemental Figure 1: Distribution of daily pulse intake by sociodemographic characteristics (n=46,939)

Supplemental Table 1: Demographic characteristics of study participants, 1999-2018 (n=46,939)

Characteristic	n	Percent (95% CI)
Age, y	46,939	100.0
20-30	8,221	20.21 (19.4-21.04)
31-50	15,731	37.32 (36.38-38.27)
51-70	15,192	31.12 (30.28-31.97)
70+	7,795	11.35 (10.87-11.85)
Gender	46,939	100.00
Male	23,472	49.15 (48.58-49.72)
Female	23,467	50.85 (50.28-51.42)
Income-to-poverty ratio	46,939	100.00
0-0.74	5,164	8.24 (7.68-8.83)
0.75-1.30	7,953	12.10 (11.41-12.82)
1.31-1.99	6,989	12.36 (11.82-12.93)
2.00-3.99	11,658	26.63 (25.72-27.56)
4.00+	11,173	33.65 (32.32-35.02)
Missing	4,002	7.01 (6.48-7.59)
Educational attainment	46,939	100.00
Less than high school	12,599	17.09 (16.24-17.98)
High school or equivalent	10,966	24.29 (23.49-25.11)
Some college	13,294	31.16 (30.38-31.94)
College graduate	10,021	27.39 (26.06-28.76)
Missing	59	0.08 (0.05-0.11)
Race-ethnicity	46,939	100.00
Non-Hispanic white	21,088	68.57 (66.49-70.57)
Non-Hispanic black	9,920	11.22 (10.13-12.4)
Mexican-American	8,083	8.10 (7.1-9.24)
Other[1]	7,848	12.11 (11.13-13.17)

Sample sizes are unweighted.

[1]Includes other Hispanic, non-Hispanic Asian, and multi-racial.

Supplemental Table 2: Association between usual pulse intake and cardiometabolic disease prevalence, without adjustment for Body Mass Index (n=46,939)

Prevalence outcome	OR (95% CI)1	P
Model 12		
CMD	0.89 (0.74-1.08)	0.239
CVD	0.71 (0.57-0.88)	0.002
CHD	0.73 (0.59-0.92)	0.007
Stroke	0.60 (0.39-0.92)	0.019
Diabetes	1.07 (0.87-1.32)	0.506
Model 23		
CMD	0.80 (0.66-0.97)	0.021
CVD	0.76 (0.61-0.95)	0.015
CHD	0.79 (0.62-0.99)	0.041
Stroke	0.64 (0.42-0.98)	0.040
Diabetes	0.87 (0.71-1.07)	0.177
Model 34		
CMD	0.79 (0.65-0.95)	0.013
CVD	0.82 (0.66-1.02)	0.072
CHD	0.81 (0.64-1.03)	0.085
Stroke	0.76 (0.49-1.18)	0.221
Diabetes	0.80 (0.64-0.99)	0.042

[1]Odds ratio associated with every once ounce-equivalent increase in usual pulse intake.

[2]Adjusted for age and sex.

[3]Addjusted for Model 1 + sociodemographic and behavioral risk factors.

[4]Adjusted for Model 2 + diet risk variables.

CMD, Cardiometabolic disease

CVD, Cardiovascular disease

CHD, Coronary Heart disease

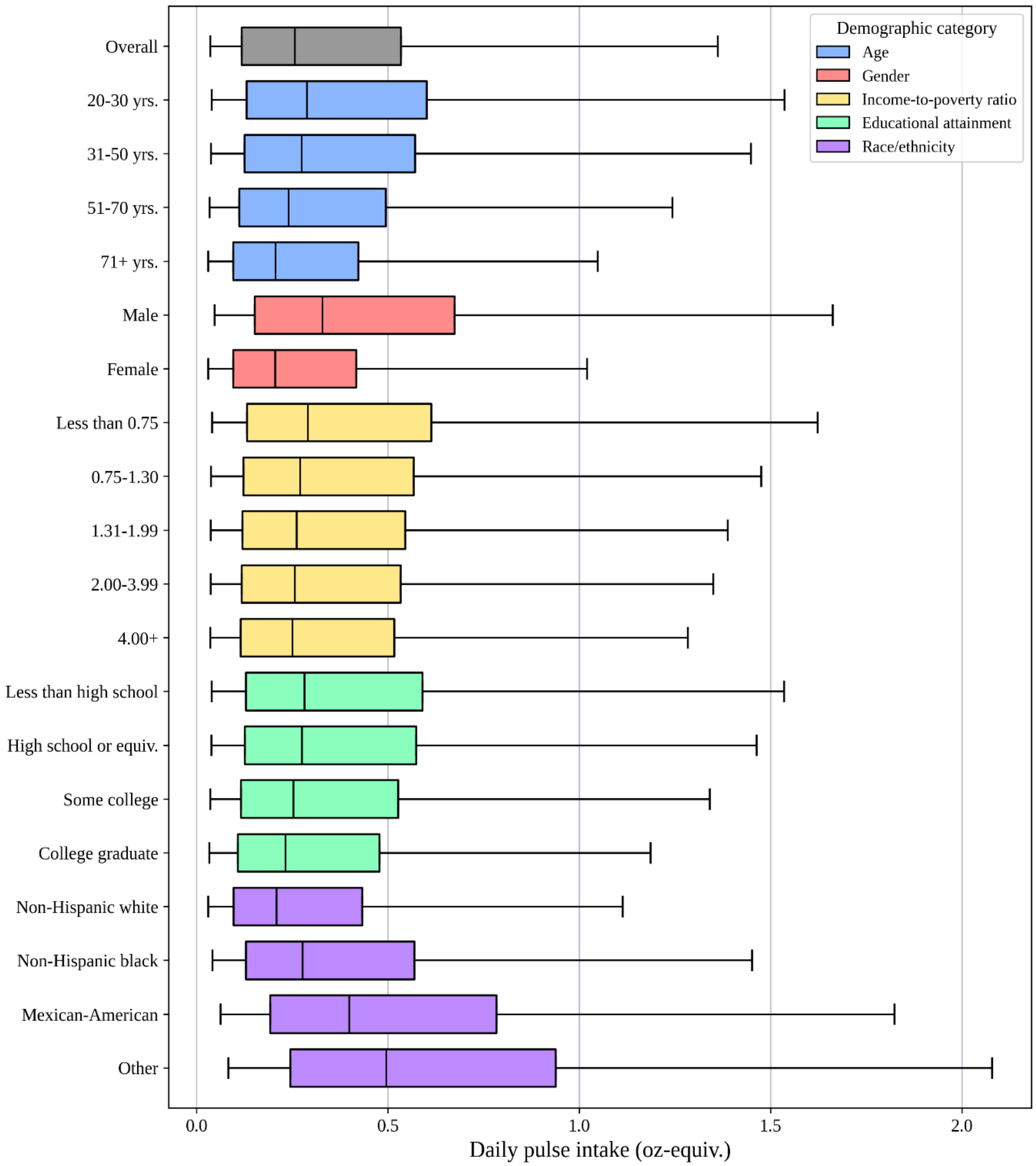

Supplemental Figure 1: Distribution of daily pulse intake by sociodemographic characteristics (n=46,939)

Within each row, bars indicate 5th, 25th, 50th, 75th, and 95th percentiles.
